# Two-year post-distraction cartilage-related structural improvement is accompanied by increased serum full-length SIRT1

**DOI:** 10.1186/s13075-024-03342-5

**Published:** 2024-05-24

**Authors:** Miya Marco, Mylène Jansen, Goran van der Weiden, Eli Reich, Yonathan H. Maatuf, Simon C. Mastbergen, Mona Dvir-Ginzberg

**Affiliations:** 1 Laboratory of Cartilage Biology, Institute of Bio-Medical and Oral Research, Faculty of Dental Medicine, Hadassah-Hebrew University of Jerusalem, P. O. Box 12272, Jerusalem, 9112102 Israel; 2 Rheumatology & Clinical Immunology, University Utrecht, University Medical Center Utrecht, Utrecht, The Netherlands

**Keywords:** Full-length Sirt1, ELISA, Joint distraction, Osteoarthritis, Serum biomarker, Endotype

## Abstract

**Background:**

Previously, fragments from Sirtuin 1 (SIRT1) were identified in preclinical and clinical samples to display an increase in serum levels for N-terminal (NT) SIRT1 vs. C-terminal (CT) SIRT1, indicative of early signs of OA. Here we tested NT/CT SIRT1 levels as well as a novel formulated sandwich assay to simultaneously detect both domains of SIRT1 in a manner that may inform us about the levels of full-length SIRT1 in the circulation (flSIRT1) of clinical cohorts undergoing knee joint distraction (KJD).

**Methods:**

We employed an indirect ELISA assay to test NT- and CT-SIRT1 levels and calculated their ratio. Further, to test flSIRT1 we utilized novel antibodies (Ab), which were validated for site specificity and used in a sandwich ELISA method, wherein the CT-reactive served as capture Ab, and its NT-reactive served as primary detection Ab. This method was employed in human serum samples derived from a two-year longitudinal study of KJD patients. Two-year clinical and structural outcomes were correlated with serum levels of flSIRT1 compared to baseline.

**Results:**

Assessing the cohort, exhibited a significant increase of NT/CT SIRT1 serum levels with increased osteophytes and PIIANP/CTX-II at baseline, while a contradictory increase in NT/CT SIRT1 was associated with less denuded bone, post-KJD. On the other hand, flSIRT1 exhibited an upward trend in serum level, accompanied by reduced denuded bone for 2-year adjusted values. Moreover, 2 year-adjusted flSIRT1 levels displayed a steeper linear regression for cartilage and bone-related structural improvement than those observed for NT/CT SIRT1.

**Conclusions:**

Our data support that increased flSIRT1 serum levels are a potential molecular endotype for cartilage-related structural improvement post-KJD, while NT/CT SIRT1 appears to correlate with osteophyte and PIIANP/CTX-II reduction at baseline, to potentially indicate baseline OA severity.

**Supplementary Information:**

The online version contains supplementary material available at 10.1186/s13075-024-03342-5.

## Introduction

In recent years, there have been many reports related to Silent Information Regulator 2 type 1 (SIRT1) as a biomarker for numerous diseases or conditions [1–5]. Many of these reports are built on previous grounds linking Sirt1 activity with health span and longevity [6–9]. As such, reduced enzymatic activity of SIRT1 has been mechanistically linked to numerous age-related diseases such as Alzheimer [2, 10], Parkinson [5], type 2 Diabetes [11], Metabolic syndrome [12], and Obesity [3, 13]. As a biomarker, SIRT1 exhibited a reduced serum level in Fatty liver disease, Obesity, Frailty, Parkinson, and Alzheimer’s disease, while an increased trend was detected in Anorexia nervosa [1–5]. Cumulatively, much of the reports with human cohorts show a striking decline in serum SIRT1 levels with the progression of these age-linked diseases [1–5, 10−13]. SIRT1 is a deacetylase protein usually confined to the nuclear or cytoplasmic compartment [6, 14], yet its shedding from the cells may be indicative of cell damage and disease development [6, 15], making this measure a powerful informer of various pathologies.

One of the most prevalent diseases related to aging is Osteoarthritis (OA), which affects approx. 500 million individuals worldwide and presents a significant socio-economic burden and risk to the quality of life [15–17]. During OA, the joint structure is significantly compromised as loss of articular cartilage combined with chronic local joint inflammation exacerbates structural damage of the joint, including osteophyte formation and subchondral bone lesions, which are part of the structural hallmarks associated with disease progression [18–21]. Loss of cartilage is one of the most characterized features of OA, and it is accompanied with dysregulation of various signaling pathways including pro-inflammatory, BMP/TGF, and WNT signaling pathways, which have been shown to evoke a pro-catabolic switch [18–21]. In particular, pro-inflammatory signaling mediated by IL1β and/or TNFα induces a significant pro-catabolic response, partly due to their effect on SIRT1 activity. SIRT1 has been established to play a critical role in maintaining cartilage integrity, partly by promoting survival [8–22, 23] and anabolic gene expression [6–8, 22, 24–28]. Mechanistically, we found that SIRT1 loses its enzymatic activity during joint inflammation by cathepsin B cleavage of the SIRT1 C-terminal domain. This event generates an enzymatically inactive 75kD polypeptide (75SIRT1), which bears an intact NT-domain [23], while the CT polypeptide may be less stable due to Sumoylation and subsequent proteolytic cleavage [23]. As such, it is conceivable that inflammatory insult of articular cartilage would generate more NT vs. CT SIRT1 variants in sera. To test this hypothesis, we previously established an indirect enzyme-linked immunosorbent assay (ELISA) to detect SIRT1 fragments in sera, based on the recognition of the N-terminal and the C-terminal SIRT1 domains in the blood (Batshon et al., [29]) and subsequently determine serum NT/CT SIRT1 ratio [29]. Accordingly, a ratio greater than one will indicate an increase in NT variant level, generated because of cathepsin B cleavage, which was associated with increased OA severity. Using this method, we found an increased NT/CT Sirt1 ratio in the blood of early OA preclinical models, following age- or post-traumatic OA induction. Moreover, genetic ablation of Sirt1 in cartilage within these models completely abrogated NT/CT Sirt1 serum levels, supporting that these fragments are derived, at least in part, from damaged cartilage [29]. Finally, preliminary data from a small human cohort exhibited that the NT/CT SIRT1 ratio is increased in early OA compared to non-OA individuals, using the Outerbridge scoring method [29], while the knowledge about serum full-length SIRT1 (flSIRT1) within this context is yet to be determined. To elucidate the dynamics of serum flSIRT1, we formulated a sandwich ELISA to detect both NT and CT domains of SIRT1 in one assay. This approach to detect the NT and CT fragments of SIRT1, likely recapitulates the presence of a full-length SIRT1 variant, while other detection assays often target one protein domain [2–4]. Here we monitored both flSIRT1 and NT/CT SIRT1 in the serum of OA patients treated with knee joint distraction (KJD) [30–32].

## Materials and methods

### Patient cohort

Serum samples obtained in two longitudinal studies of patients treated with 6 weeks of KJD were assessed [33]. OA patients knee with Kellgren-Lawrence grade > 2 and age < 65 years old (*n* = 43) were included in 2 separate randomized controlled trials (RCTs): (1) patients considered for high tibial osteotomy (HTO) randomized to either HTO or KJD (*n* = 23 KJD patients), and (2) patients considered for total knee arthroplasty (TKA) randomized to either TKA or KJD (*n* = 20 KJD patients). This study included only patients treated with KJD surgery in these RCTs. Full inclusion and exclusion criteria, as well as treatment details, have been published previously [34].

Both original trials were granted ethical approval by the medical ethical review committee of the University Medical Center Utrecht (protocol numbers 10/359/E and 11/072), registered in the Netherlands Trial Register (trial numbers NL2761 and NL2680), and were performed in accordance with the ethical principles of the Declaration of Helsinki. All patients provided written informed consent.

### Data collection

Serum samples were collected at baseline (baseline; T0), and after 24 months (T2) KJD surgery. Collected serum samples were analyzed for NT/CT SIRT1 and flSIRT1 according to the methods described below. Further, serum from baseline and 2-years were analyzed for systemic collagen type-II markers, specifically serum N-propeptide of type IIA procollagen (PIIANP; Linco, EZPIIANP-53 K) for synthesis and urinary C-telopeptide of type-II collagen (CTXII; Cartilaps; corrected for urine creatinine) for breakdown; normalized Z-scores were calculated to express the net collagen type-II synthesis as a Z-index (Z_PIIANP_ − Z_CTXII_) [34]. Additionally, at baseline and 24 months, patients filled out Knee Injury and Osteoarthritis Outcome Score (KOOS), visual analogue scale (VAS) pain questionnaires, MRI scans and weight-bearing radiographs of the treated knee were performed. From radiographs the most affected compartment (MAC; medial or lateral) was determined and they were analyzed using KIDA software to obtain a minimum joint space width (JSW; mm), mean JSW of the MAC (mm), osteophyte size (mm^2^), and subchondral bone density of the MAC (mm Aluminum equivalent, in reference to an aluminum step wedge) [37]. From the MRI scans, the mean cartilage thickness (ThCtAB; mm) and percentage of denuded bone area (dABp; %) were determined in the MAC [38].

### NT/CT SIRT1 indirect enzyme-linked immunosorbent assay (ELISA)

Indirect ELISA was carried out for KJD serum samples to detect NT/CT SIRT1 fragments, according to the method in Batshon et al., [29]. Diluted serum samples (1:2000) were used against two standard curves in two separate opaque plates; CT reactive standard curve with a full-length human SIRT1 protein ranging from 0.2 to 100 ng/mL (i.e. CT plate); or an NT reactive standard curve with human 75SIRT1 ranging from 0.2 to 100 ng/mL (i.e. NT plate). NT-SIRT1 reactive (Millipore #07-131; 1:2000) or CT-SIRT1 reactive (Bethyl Laboratories #A300-688; 1:2000 both in blocking solution containing PBS with 1.5% BSA and 0.05% Tween 20). Diluted serum (1:2000) and standard curves were allowed to adhere and incubated the next day with blocking for 3 h. After three PBS-T washes samples were incubated with primary antibody overnight. Following three washes, the plate was incubated with a secondary anti-rabbit HRP antibody (Cat# ab7090, Abcam, UK) for 1 h at room temperature and washed five times with PBS-Tween. Substrate signal was developed using TMB (Cat# 0410 Southern Biotech, AL, US) for the NT-plate or SuperSignal ELISA Femto (Thermo Fischer Scientific) kit for the CT plate. Data were analyzed using TECAN infinite M200PRO software (450 nm)and an NT/CT SIRT1 ratio calculated, following standard curve comparisons.

### Formulation of NT- and CT-Reactive Antibodies for SIRT1

Before formulating the flSIRT1 ELISA detection methos, we generated domain specific antibodies. Mouse monoclonal antibodies to detect human CT SIRT1 domain were generated, using injected polypeptides corresponding to the aa 700–747 (i.e.CT peptides; Proteogenix, France). Similarly, a rabbit-derived polyclonal antibody was generated using a polypeptide corresponding to the human NT SIRT1 aa 1-131 (i.eNT peptides; Adar Biotech, Rehovot Israel). Monocolonal CT-reactive (*mCT*) antibodies, and polyclonal NT reactive antibodies (pNT) were isolated, and affinity purified using Protein-G resin (2–3 mL bed-volume, Cat. No. L00209, Genscript Biotech, Singapore). Following TBS wash, antibodies were eluted by 0.1 M glycine-HCL buffer pH 2.8 and pH was adjusted by 1 M CaCo3 pH 9.6, to about pH 6.

### Monoclonal antibody (mCT) affinity preference assay

To determine antibody specificity of mCT monoclonal antibodies, a sandwich ELISA method was employed using the -NT or -CT peptides. Briefly, 100 µl/well of mCT in the concentration of 1:20,000 diluted with coating buffer: (pH = 9.5; 44mM Sodium bicarbonate and 6mM Sodium carbonate) and incubated in an opaque ELISA 96 well plate (Nunc F96 MAXISORP, cat #442,404) for 2 h at room temperature. Following three washes with 0.05% PBS-Tween 20, antibodies were blocked with PBS solution containing 1.5% BSA and 0.05% Tween 20 (blocking solution) for 2 h at room temperature. Next, the wells were washed three times and incubated with 200 ng/mL of -NT or -CT peptides in PBS (Sigma Aldrich) overnight at 4 C°. The following day, after three additional PBS-T washes, either NT-SIRT1 reactive (Millipore #07-131; 1:2000) or CT-SIRT1 reactive (Bethyl Laboratories #A300-688; 1:2000 both in blocking solution) were added (100 µl/well) and incubated 2 h at room temperature then washed four times. Finally, the plate was incubated with a secondary anti-rabbit HRP antibody (Cat# ab7090, Abcam, UK; 1:20,000) for 1 h at 37 C°, washed five times and signal developed using TMB (Cat# 0410 Southern Biotech, AL, US) for 15–30 min at room temperature, followed by 12 M HCL stop buffer. Plates were read using TECAN infinite plate reader and M200PRO software (450 nm).

### Polyclonal antibody (pNT) affinity preference assay

To assess pNT affinity to NT or CT SIRT1 domains, we employed an indirect ELISA method adapted from Batshon et al., [29] using the -NT or -CT peptides. Briefly, 200 ng/mL of NT or CT peptides were diluted in PBS (Sigma Aldrich) and incubated in an transparent ELISA 96 well plate (Nunc F96 MAXISORP, #442,404) overnight at 4 C°. Next day, peptides were blocked with blocking solution for 3 h, washed three times with PBS-Tween and incubated overnight at 4^o^C with pNT antibody (1:1,000, blocking buffer). Following three washes, the plate was incubated with a secondary anti-rabbit HRP antibody (Cat# ab7090, Abcam, UK) for 1 h at 37 C°, and washed five times with PBS-Tween. The substrate signal was read using as previously indicated.

### Assessing antibody specificity via Immunoblot analysis

A pcDNA-Flag-His-SIRT1 expression plasmid for human full-length (fl.) SIRT1 was a kind gift of Prof. Danny Rienberg (New York University). Based on this construct, human cleaved SIRT1 plasmids encoding the 75SIRT1 protein (i.e., a.a.1-534 of SIRT1; approx. 75 kDa in molecular weight of the translated protein) were designed [29]. Protein isolation of standards was carried out according to Batshon et al., [29]. Briefly, SIRT1 plasmids were transfected into HEK293 cells using a GenJet™ DNA In Vitro Transfection *Reagent* (SignaGen Laboratories). Cell lysates were obtained in 20 mM Tris-HCl, 250 mM NaCl, 10 mM Imidazole, pH 8.0, buffer, which was supplemented with a protease inhibitor cocktail and underwent three freeze-thaw cycles. His-tagged proteins were loaded on Ni-NTA columns and using an imidazole gradient (20 mM Tris-HCl, 250 mM NaCl, 250 mM Imidazole, 10% glycerol, pH 7.2). The eluted fractions were examined for purity using the Bradford assay and Coomassie blue staining for SDS-PAGE gels. Protein fractions were pooled and concentrated using a 30-kDa cutoff Amicon filter (Merck-Millipore). In-vitro cleavage assay was performed with flSIRT1 with h. Cathepsin B, as previously described (Kumar et al.,) [35]. The cathepsin B inhibitor, CA074me (2 µM) was used to block cathepsin B-mediated cleavage.

For western blotting, in-vitro protein extracts were run on standard 10% SDS PAGE gels and finally transferred to PVDF membranes. The immunoblots were incubated with mCT (1:1000) or pNT (1:1000), while secondary antibodies were Anti-mouse alkaline-phosphatase (AP)-conjugated (Sigma-Aldrich, St Louis, MO, Cat# A3562), Anti-rabbit AP-conjugated (Sigma-Aldrich, St Louis, MO, Cat# A3687).

### Formulation of flSIRT1 Sandwich ELISA assay

**Fig. 1:**
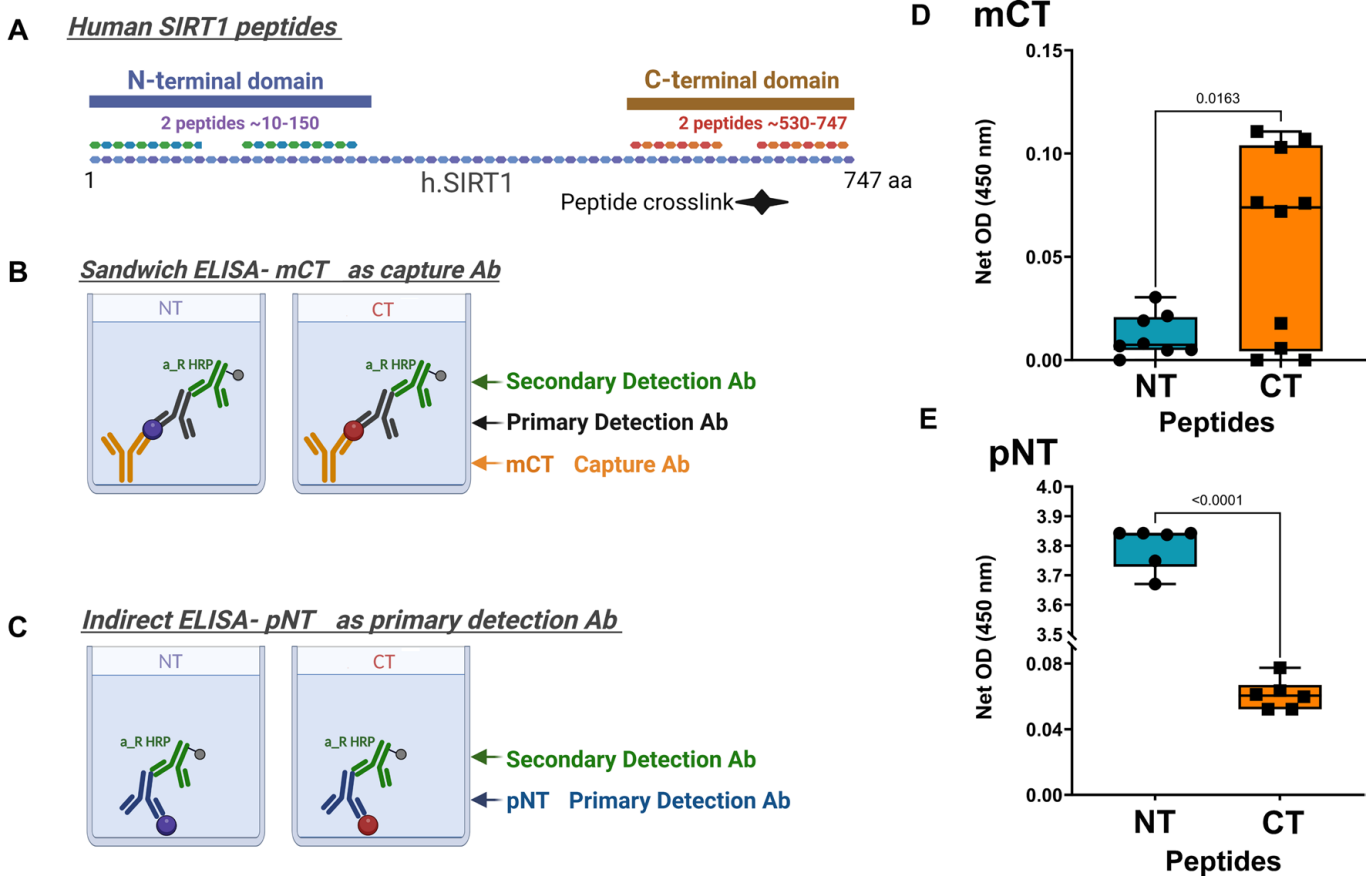
Antibody design and site-specific affinity binding: **(A)** The full-length SIRT1 protein (1-747aa) is illustrated with its N-terminal domain (blue rectangle), and C-terminal domain (brown rectangle). We designed peptides corresponding the NT a.a. SIRT1 sequence (composed of 2 peptides spanning ∼ 10-150aa) and the CT SIRT1 sequence (composed of 2 peptides spanning ∼ 530–747). To generate CT antibodies (mCT), CT peptides were injected to mice, while NT peptides were injected to rabbits to generated polyclonal NT-reactive antibody (pNT). **(B)** Right panel illustrates the sandwich ELISA method, wherein the NT fragments were detected with primary NT detection Ab (Millipore 07-131 or “a”; 1:2,000), following capture with mCT (*n* = 8). To detect CT (red ball) or NT (purple ball) peptides, we employed a sandwich ELISA, wherein mCT served as capture and Bethyl (CT -reactive; A300-688) served as a detection antibody. **(C)** Indirect ELISA was adapted from Batshon et al., 2020 [29]. Left scheme illustrates NT-peptide coated wells, incubated with pNT (1:1,000). Right panel indicates CT-peptide coated wells incubated with pNT (*n* = 6). **(D)** The net OD was detected for sandwich ELISA in panel B to assess the reactivity of mCT to the NT vs. CT peptide. Statistical significance determined by Unpaired T-test of net signal, two-tailed, P value = 0.0201. The net OD was detected for indirect ELISA in panel C to determine the specificity of pNT to NT or CT peptides. Statistical analysis was carried out using Mann-and-Whitney, assuming significance at *p* < 0.05

Sandwich ELISA was performed by adsorbing 100 µl/well mCT antibody in a concentration of 1:20,000 diluted with coating buffer and incubated in an ELISA 96 well plate (Nunc F96 MAXISORP, #442,404) for 2 h at room temperature and then washed three times with 200 µl/well of 0.05% PBS-Tween 20. Next, serum (diluted 1:2000 in PBS) or human SIRT1 standard (Cat# 7714-DA, R&D systems, MN; standard range 100-3.125ng/mL) was added and incubated overnight, 4^O^C, followed by blocking for 2 h, and three more washes. Next, 100 µl/well pNT (1:1000 in blocking buffer) were added and incubated 2 h at room temperature. Finally, after four washes the plate was incubated with a secondary anti-rabbit HRP antibody (Cat# ab7090, Abcam, UK) for 1 h at 37 C°, washed five last times and the signal was read as previously described. A schematic representation of the antibody reactivity and ELISA setup is presented in Fig. 1A-C.

To validate if the sandwich ELISA protocol can detect only flSIR1, three different antigen assays were executed using descending concentrations (100, 50, 25, 12.5, 6.25, 3.125 ng/mL) of; (i) flSIRT1, (ii) 50% flSIRT1 + 50% 75SIRT1; (iii) 75SIRT1. The standard curve was plotted and assessed for linearity using Pearson’s correlation assay.

To assess the technical robustness of the flSIRT1 sandwich ELISA assay, three validation tests were performed: Dilution recovery, percent coefficient of variance (%CV), *Spike in and recovery, as detailed below (i-iii) and summarized in ****SD1 ****table.*

(i) *dilution recovery*- provide information about the precision of assay results for samples tested at different dilutions (range in which the analyte dose-response graph is linear).
  $${\rm{\% Linearity = }}{{{\rm{Previous}}\,{\rm{observed}}\,{\rm{value}}\,{\rm{in}}\,{\rm{the}}\,{\rm{dilution}}\,{\rm{series}}} \over {{\rm{(Observed}}\,{\rm{Concentration}}\,{\rm{\times}}\,{\rm{2 )}}}} \times {\rm{100 }}.$$
   The mean percent linearity for each should be 80–120% [36].
(ii) *The percent coefficient of variance (%CV)* – The ratio of the standard deviation (σ) of a set of measurements to its mean (µ). %CV is a general guideline to gauge the overall reliability of our immunoassay results, indicating any inconsistencies or inaccuracies in ELISA results. To express the precision of immunoassay results, we used two types of this analysis: inter-assay %CV (a measure of the variance between runs of sample replicates on different plates that can be used to assess plate-to-plate consistency) of less than 15% while intra-assay %CV (a measure of the variance between data points within the same plate) should be less than 10%. Larger variance indicates greater inconsistency and error [37].
(iii) *Spike in and recovery-* is used to determine whether analyte detection is affected by a difference between the diluent used to prepare the standard curve and the biological sample matrix, wherein an acceptable recovery range of 80–120% is recommended [34].

### Statistical analysis

As several parameters were not normally distributed, Spearman correlations were used to evaluate the association between baseline NT/CT SIRT1 or flSIRT1 with all baseline clinical and structural parameters individually. Similarly, Spearman correlations were used to assess the association between two-year changes in NT/CT SIRT1 or flSIRT1 with two-year changes in clinical and structural parameters. To further evaluate the association of NT/CT SIRT1 and flSIRT1 with structural treatment response, patients were scored with two structural response scores: (1) 0–4 based on how many cartilage-related parameters (min JSW, MAC JSW, ThCtAB, dABp) displayed an improvement over two years, and (2) 0–5 based on improvement in cartilage-related parameters and subchondral bone density. Spearman correlations were performed between these scores and the two-year change in NT/CT SIRT1 or flSIRT1; for these analyses, only patients with all 4 or 5 measures available at both baseline and two years were included.

GraphPad Prism software for graphical illustration and IBM SPSS Statistics for statistical analysis was used; a p-value of < 0.05 was considered statistically significant. Illustrations and schemes were generated via BioRender software.

## Results

### Patients

**Fig. 2:**
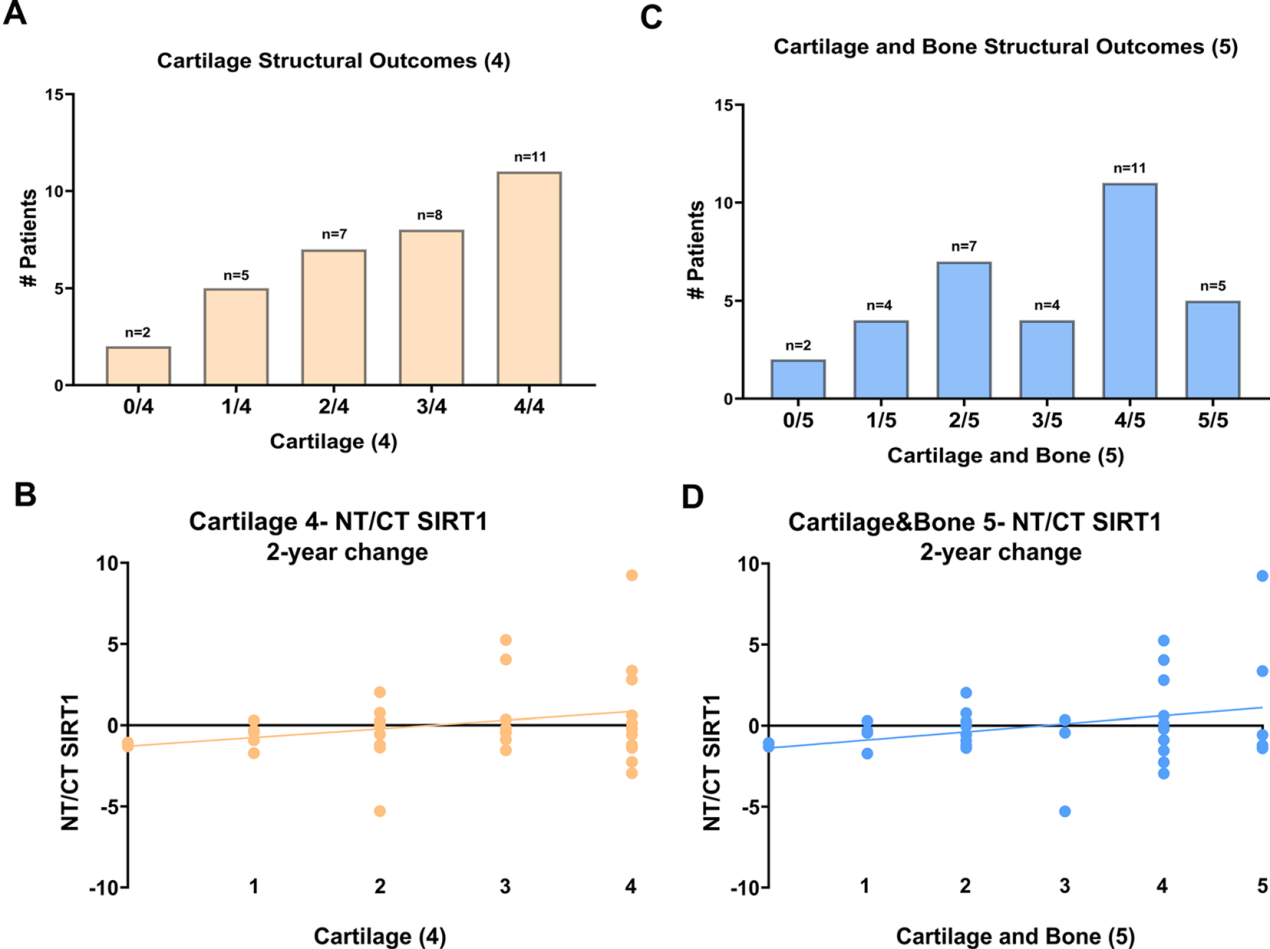
NT/CT SIRT1 changes in association with KJD structural response: Joint distraction cohort (*n* = 33 exhibiting all parameters) were stratified for the number of patients showing 2-year improvement post-distraction for 4 cartilage structural variables (Mean MAC JSW; Min JSW; MAC ThCtAB and dABp) displayed in (**A**) histogram, reflecting a low structural response (0/4) vs. a high structural response (4/4). The correlation of these four patient categories for two years changes is plotted in panel B **(**ρ = 0.8, *p* = 0.133**)**. Joint distraction cohort (*n* = 33) were stratified for the number of patients showing two-year improvement post-distraction for 5 cartilage and bone structural variables (Mean MAC JSW; Min JSW; MAC ThCtAB, dABp and MAC_Bone density) displayed in (**C**) histogram, reflecting a low structural response (0/5) vs. a high structural response (5/5). The correlation of these five patient categories for two years changes is plotted in panel (**D) (**ρ = 0.685, *p* = 0.132**)**. Statistical significance was determined using a two-tailed Spearman r correlation, assuming significance at *p* < 0.05

Of the total 43 patients treated with KJD, 36 patients could be analyzed. Seven were excluded due to incomplete data or sample collection. Baseline characteristics, baseline clinical and structural parameters, and their two-year changes are shown in Table 1. The structural response scores on either cartilage-related parameters (0–4) or cartilage and bone-related parameters (0–5) are displayed in Fig. 2A, and 2C respectively (*n* = 33).

**Table 1:** Knee joint distraction patients baseline characteristics and two-year changes

Parameter	Baseline	Two-year change
Age, years	54.1 ± 6.6	
Male gender, n (%)	22 (61)	
BMI, kg/m^2^	27.1 ± 13.4	
Kellgren-Lawrence grade, n (%)		
- Grade 0	0 (0)	
- Grade 1	5 (14)	
- Grade 2	5 (14)	
- Grade 3	15 (42)	
- Grade 4	11 (31)	
Total KOOS, 0-100	47.9 (28.2–57.0)	25.5 (12.4–38.1)
VAS pain, 10 − 0	6.3 (3.7–7.5)	-2.9 (-4.8 – -1.1)
MAC ThCtAB, mm	1.9 (1.4–2.6)	0.0 (-0.1–0.3)
MAC dABp, %	23.9 (11.8–38.8)	-0.8 (-8.4 – -1.4)
MAC mean JSW, mm	1.8 (0.6–2.9)	0.3 (-0.1–1.1)
Minimum JSW, mm	0.0 (0.0–0.5)	0.4 (0.0–1.0)
MAC subchondral bone density, mm Al eq	37.1 (35.0–39.1)	1.6 (-0.4–4.8)
Osteophyte area, mm^2^	37.5 (20.1–56.0)	5.7 (1.2–13.5)
NT/CT, ratio	7.0 (3.5–11.3)	-0.4 (-1.3–0.5)
flSIRT1, µg/mL	9.8 (2.0–17.2)	-0.6 (-9.3–5.9)
Z-index PIIANP/CTX-II	0.1 (-0.7–0.9)	0.5 (0.0–1.5)

Mean ± standard deviation, median (Q1 – Q3), or n (%) are presented. BMI: body mass index; KOOS: knee injury and osteoarthritis outcome score; VAS: visual analogue scale; MAC: most affected compartment; ThCtAB: mean cartilage thickness over the total subchondral bone area; dABp: percentage of denuded subchondral bone area; JSW: joint space width; mm Al eq: mm Aluminum equivalent (in reference to an aluminum step wedge); NT/CT: N-terminal/C-terminal; flSIRT1: full-length sirtuin-1; PIIANP: N-propeptide of collagen IIA; CTX-II: C-terminal cross-linked telopeptides of type II collagen

### NT/CT SIRT1 associated with clinical and structural markers

At baseline, higher NT/CT SIRT1 was significantly correlated with larger osteophytes (ρ = 0.350; *p* = 0.036) and lower collagen type-II Z-index (ρ=-0.363; *p* = 0.041) as seen in Table 2. Other baseline parameters were not significantly correlated with NT/CT SIRT1, though in general higher NT/CT SIRT1 serum values at baseline, seemed to indicate more severe OA (Table 2).

**Table 2:** Baseline and 2-year change correlations for serum NT/CT SIRT1 and clinical or structural markers

Parameter	ρ	*P*-value
**Baseline**		
KOOS	−0.058	0.736
VAS pain	0.105	0.541
MAC ThCtAB	−0.172	0.337
MAC dABp	0.220	0.211
MAC JSW	−0.242	0.155
Min JSW	−0.214	0.210
MAC bone density	−0.126	0.464
Osteophytes	0.350	**0.036**
Z PIIANP/CTX-II	−0.363	**0.041**
**Two-year changes**		
KOOS	0.210	0.226
VAS pain	−0.182	0.288
MAC ThCtAB	0.201	0.262
MAC dABp	−0.331	0.056
MAC JSW	0.005	0.976
Min JSW	0.030	0.862
MAC bone density	0.086	0.616
Osteophytes	−0.010	0.956
Z PIIANP/CTX-II	0.321	0.078

Spearman correlation coefficients and p-values are presented. NT/CT SIRT1: N-terminal/C-terminal sirtuin-1; BMI: body mass index; KOOS: knee injury and osteoarthritis outcome score; VAS: visual analogue scale; MAC: most affected compartment; ThCtAB: mean cartilage thickness over the total subchondral bone area; dABp: percentage of denuded subchondral bone area; JSW: joint space width; PIIANP: N-propeptide of collagen IIA; CTX-II: C-terminal cross-linked telopeptides of type II collagen

For two-year changes, a higher increase in NT/CT SIRT1 seemed to correspond with increased improvement after treatment, although no correlations were statistically significant (Table 2). Specifically, 2-year adjusted values of NT/CT SIRT1, only MAC dABp (ρ=-0.331; *p* = 0.056) and Z-index (ρ = 0.321; *p* = 0.078) showed a marginal tendency towards association. However, Spearman correlation coefficients exhibited a reversed trend for 2-year changes in all parameters, potentially indicating that this biomarker cannot reflect a response to treatment but can potentially indicate baseline clinical variations in OA severity reflected by osteophytes and increased collagen type-II processing. These data are in line with previous work showing that this biomarker may indicate a worsening of OA severity [29].

### NT/CT SIRT1 changes compared with the extent of KJD structural response

Figure 2 shows the correlations between two-year changes in NT/CT SIRT1 and cartilage structural response scores (Fig. 2B), as well as cartilage and bone structural response scores (Fig. 2D). Neither correlation was statistically significant (Fig. 2B: ρ = 0.8 with *p* = 0.133 and Fig. 2D ρ = 0.685 with *p* = 0.132, respectively).

Overall, while we expected to observe reduced NT/CT SIRT1 correlated with improved structural variables following 2 years, this association was not established for NT/CT SIRT1 (Table 2). Moreover, we did establish a significant association of NT/CT SIRT1 with the baseline appearance of osteophytes, and collagen type-II turnover. Nonetheless, the similar increase in NT/CT SIRT1 for 2-year structural improvement, indicates that NT/CT SIRT1 may not serve as a biomarker for response to distraction. To this end, we next sought to monitor changes in serum full-length SIRT1 (fl.SIRT1) only, as an alternative approach.

### Formulating a new method for the detection of serum full-length SIRT1

To formulate a new method to detect full-length SIRT1 in serum, we generated site-specific antibodies (monoclonal CT-specific denoted “mCT” and polyclonal NT-specific denoted “pNT”), and determined their specificity using the ELISA method for both antibodies against two peptides (NT peptide or CT peptides), as shown in Fig. 1A C. The data show that mCT2 preferentially recognized the CT peptides (Fig. 1D), while pNT preferentially recognized NT peptides (Fig. 1E).

**Fig. 3:**
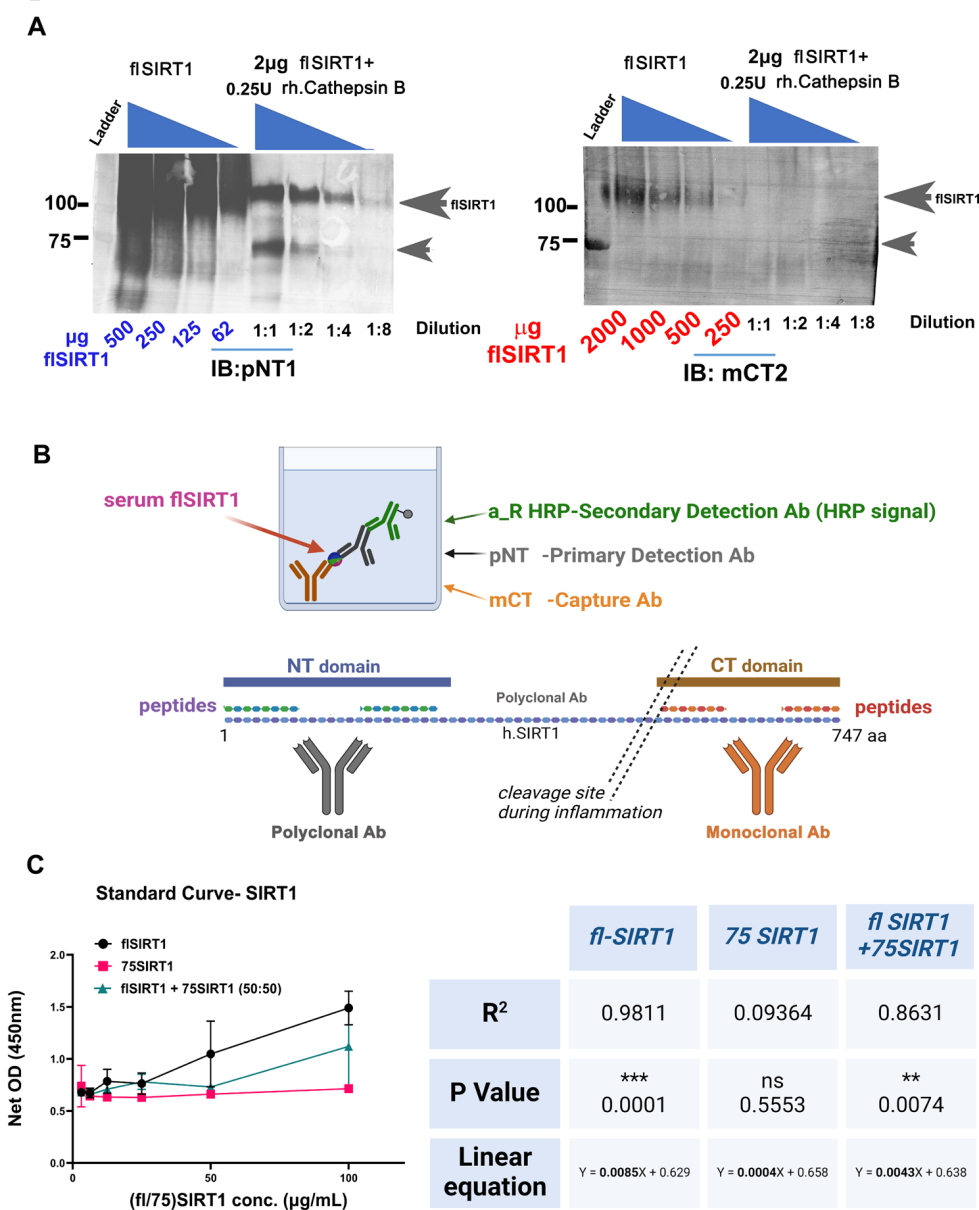
Detecting Antibody Specificity for NT and CT SIRT1 domains. (**A**) In-Vitro cleavage of SIRT1 was carried out by incubating 2µg flSIRT1 (110 kDa) with recombinant human Cathepsin B (rh. Cathepsin B), for 15 min at RT, to generate the 75SIRT1 NT-intact cleaved variant (ranging till 8-fold dilution) or loading 62–500 µg (pNT)/ 2000–250 µg (mCT) human full-length SIRT1. Each immunoblot was incubated with NT reactive (pNT) or CT-reactive (mCT) antibody. **(B)** The formulated ELISA method was designed to detect full-length SIRT1, using the sequence-specific antibodies mCT and pNT. **(C)** Sandwich ELISA was performed according to the set up in (B) with three antigens at a range of concentrations (100,50,25,12.5,6.25,3.125 ng/mL): (i) flSIRT1 (black circle), (ii) 75SIRT1 (pink circle), (iii) both flSIRT1 and 75SIRT1 at 50% v % (green rectangle). The right table exhibits the R^2^, p-value and standard curves for (i)-(iii), using Pearson’s correlation. Notably, R^2^ closer to 1 indicates a linear fit of the standard curve of the antigen to the antigen, which is seen with flSIRT1 (R^2^ = 0.9811), while 75SIRT1 R^2^ was 0.0936

Additional immunoblot assays confirmed the capacity of these antibodies to recognize their respective protein domains, as pNT was able to detect flSIRT1 and the 75 kDa cleaved variant, with descending concentrations (Fig. 3A, left immunoblot). In contrast, mCT2 only recognized the flSIRT1, with descending protein concentration (110kD), (Fig. 3A, right immunoblots). In sum, immunoblot results (Fig. 3A) confirm that pNT Ab preferentially binds to the NT domain of SIRT1, while mCT preferentially recognizes the CT domain of SIRT1.

We next formulated sandwich ELISA using mCT as adsorbed capture Ab and pNT as a primary detection antibody, to monitor only flSIRT1 sera, according to materials and method and scheme displayed Fig. 3B, for *n* = 33 of the cohort displaying all 4 or 5 structural parameters. Detection of flSIRT1 displayed a linear standard curve, while the standard curve from 75SIRT1 with or without flSIRT1 displayed undetected or reduced slops vs. the flSIRT1 epitope (Fig. 3C), indicating that the assay is capable of detecting flSIRT1.

Technical validation included %CV, spike-in and dilution recovery (SD1 table). An inter-assay (10.84%) or intra-assay (4.76%) Coefficient of variability (%CV) were obtained and are consistent with the standard expectation (i.e. lesser than *15%* and *10%*, respectively). Spike-and-recovery was calculated using 1:2000 diluted sample spiked with 100ng/mL human full-length (h.fl or fl) SIRT1, resulting in 81.6%, which relatively low yet acceptable value. Finally, dilution Recovery exhibited 80.18% for the serum dilution range of 1:1000 to 1-3000, which coincides with our previous data for NT/CT SIRT1 [29], thereby we next used a 1: 2,000–3,000 dilution range for serum for our assays.

### flSIRT1 association with clinical and structural markers

There were no statistically significant correlations between flSIRT1 and clinical or structural markers, neither at baseline nor for two-year changes (Table 3), although the two-year change in MAC dABp (ρ=-0.303; *p* = 0.082) showed a tendency towards association. While no correlations were significant, the direction of the correlations was consistent: higher flSIRT1 levels at baseline indicate less OA joint damage, and a higher two-year increase in flSIRT1 indicates more structural improvement after treatment.

**Table 3:** Baseline and 2-year change correlations for serum flSIRT1 and clinical or structural markers

Parameter	ρ	*P*-value
**Baseline**		
KOOS	0.155	0.368
VAS pain	−0.069	0.690
MAC ThCtAB	0.062	0.731
MAC dABp	−0.196	0.267
MAC JSW	−0.001	0.994
Min JSW	0.089	0.607
MAC bone density	−0.138	0.421
Osteophytes	−0.176	0.305
Z PIIANP/CTX-II	0.111	0.547
**Two-year changes**		
KOOS	0.222	0.200
VAS pain	−0.002	0.989
MAC ThCtAB	0.199	0.268
MAC dABp	−0.303	0.082
MAC JSW	0.102	0.554
Min JSW	0.011	0.948
MAC bone density	0.008	0.962
Osteophytes	−0.036	0.837
Z PIIANP/CTX-II	−0.118	0.526

Spearman correlation coefficients and p-values are presented. flSIRT1: full-length sirtuin-1; BMI: body mass index; KOOS: knee injury and osteoarthritis outcome score; VAS: visual analogue scale; MAC: most affected compartment; ThCtAB: mean cartilage thickness over the total subchondral bone area; dABp: percentage of denuded subchondral bone area; JSW: joint space width; NT/CT: N-terminal/C-terminal; flSIRT1: full-length sirtuin-1; PIIANP: N-propeptide of collagen IIA; CTX-II: C-terminal cross-linked telopeptides of type II collagen

**Fig. 4:**
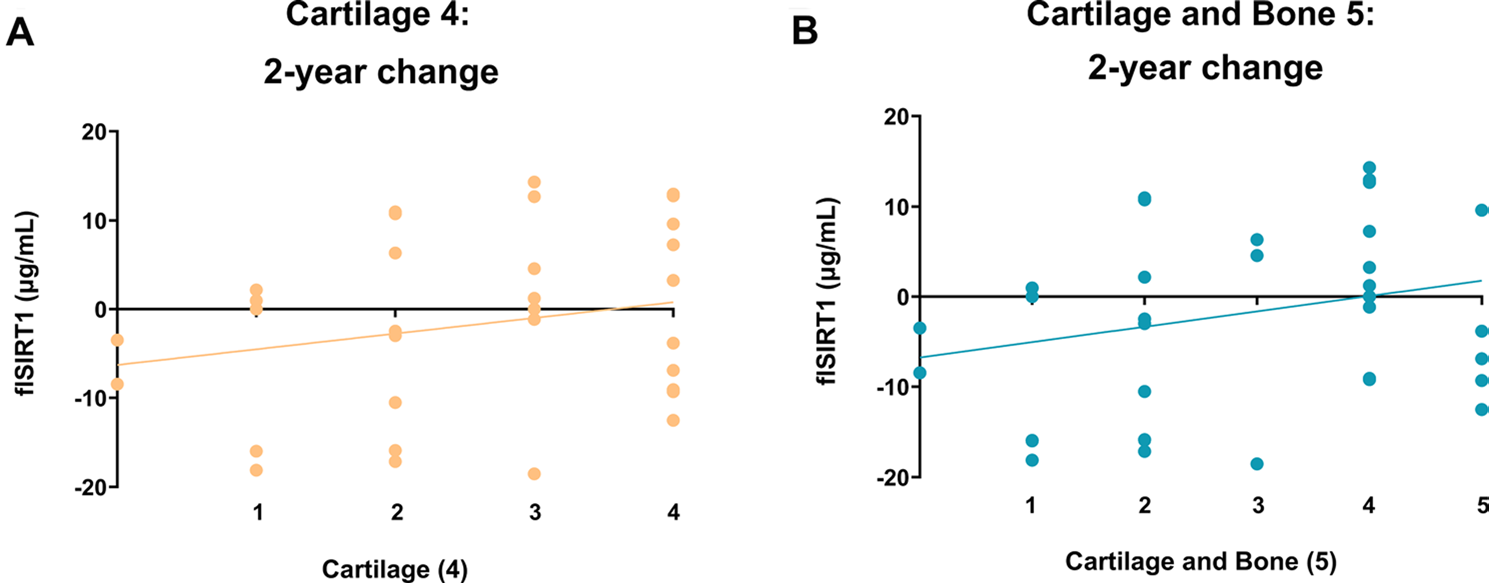
Post-distraction increase in flSIRT1 in relation to structural improvement. Joint distraction cohort (*n* = 33) were stratified for the number of patients showing two-year post-distraction improvement in 4 cartilage structural variables (Mean MAC JSW; Min JSW; MAC ThCtAB and dABp), reflecting a low structural response (0/4) vs. a high structural response (4/4). The correlation of these four patient categories for two-year changes is plotted in panel (**A**) **(**ρ = 0.8, *p* = 0.133**)**. Joint distraction cohorts (*n* = 33) were stratified for the number of patients showing 2-year improvement post-distraction, for 5 cartilage and bone structural variables (Mean MAC JSW; Min JSW; MAC ThCtAB, dABp and MAC_Bone density), reflecting a low structural response (0/5) vs. a high structural response (5/5). The correlation of these five patient categories for two years changes is plotted in panel (**B**) **(**ρ = 0.6, *p* = 0.24). Statistical significance was determined using a two-tailed Spearman r correlation, assuming significance at *p* < 0.05

Assessing response exhibited a similar upward increase in serum flSIRT1 with more cartilage response (ρ = 0.8; *p* = 0.133, Fig. 4A) which was consistent for cartilage and bone response (ρ = 0.6; *p* = 0.24, Fig. 4B), while statistically insignificant.

### Reduced MAC dABp is the most variable structural feature among KJD responders exhibiting an inverse relation to serum flSIRT1 levels

**Fig. 5:**
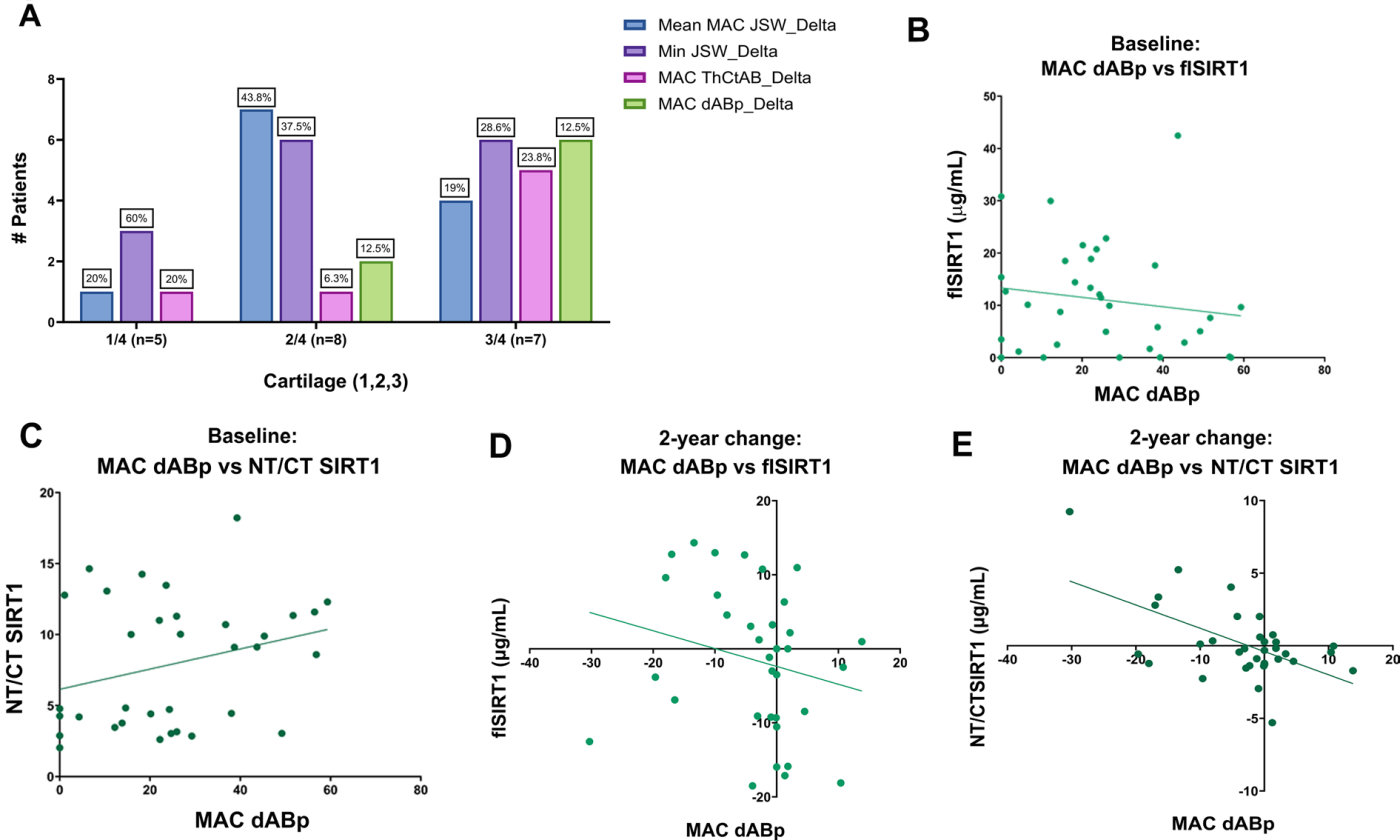
Improvement in dABp is differential among the cartilage structural response variables and is accompanied with flSIRT1 increase. Joint distraction cohort (*n* = 33) were stratified for the number of patients showing 2-year improvement post-distraction for 4 cartilage structural variables (Mean MAC JSW; Min JSW; MAC ThCtAB and dABp), reflecting a low structural response (0/4) vs. a high structural response (4/4). (**A**) The percent within a given category (i.e. 1/4, 2/4 or 3/4) that shows a change in one of the four cartilage-related variables. (**B**) Association of dABp with flSIRT1 **(**ρ= -0.196, *p* = 0.133**)**; and (**C**) NT/CT SIRT1 **(**ρ = 0.22, *p* = 0.2115**)**, at baseline. Two-year change in dABp compared to (**D**) flSIRT1 **(**ρ= -0.303, *p* = 0.0817**)**; or **(E)** NT/CT SIRT1 **(**ρ= -0.330, *p* = 0.056**)**. Statistical significance was determined using a two-tailed Spearman r correlation, assuming significance at *p* < 0.05

Next, we attempted to closely assess the KJD patient cohort to the four cartilage-related measures (Fig. 5A). On average the most prevalent structural variable improved in the patient cohort are Min JSW (av = 42.03%), Mean MAC JSW (av = 27.6%), while MAC ThCtAB (av = 16.7%) and dABp (av = 13.7%) exhibited lesser improvement. However, dABp change appears to be differentially improved amongst the 3 cartilage-response categories (i.e. 1/4 low; 2/4 moderate and 3/4 high responders), indicating that changes in dABp may reflect a high response for other structural outcomes. Interestingly, the baseline of NT/CT SIRT1 (ρ = 0.211; *p* = 0.219, Fig. 5C) and flSIRT1 (ρ= -0.196; *p* = 0.133, Fig. 5B) are weakly correlated with dABp, but show inverse trends. While an increase in NT/CT SIRT1 is associated with more denuded bone, increase in flSIRT1 reflects less denuded bone, at baseline. Both of these trends are consistent with the biochemical justification for these biomarkers. Notably, the positive slope of NT/CT SIRT1 with increased denuded bone at baseline is consistent with lower collagen type-II Z-index (ρ=-0.363; *p* = 0.041; Table 2), to support that this biomarker can provide insight related to baseline OA severity.

On the other hand, two-year changes for reduced dABp were accompanied by upward trends in NT/CT SIRT1 (Fig. 5E, ρ= -0.330, *p* = 0.056), similar to the increased flSIRT1 (Fig. 5D, ρ= -0.303, *p* = 0.082), the latter trend also seen at baseline. Overall, the increase NT/CT SIRT1 appears to reflect baseline structural damage, while the increase in flSIRT1 may reflect responsiveness to KJD through changes in dABp.

## Discussion

One of the unmet needs in the field of OA research is the need for suitable endotypes or biomarkers that may better phenotype subgroups of OA patients. This need is critical given the heterogeneity of this population and the need for personalized care. Importantly, clinical symptoms (e.g., radiographic features, pain, and joint effusion of exposed subchondral bone) typically arise only once the cartilage and surrounding tissues are already severely damaged [19–21]. OA is often detected radiographically by a decrease in joint space width (JSW), though the change is visible only after significant cartilage degeneration has taken place. While MRI imaging is more capable of effectively detecting subtle structural alterations on a tissue level, it is often inaccessible and cannot be used routinely for OA detection. Significant efforts have been invested in recent years to develop disease-modifying drugs (DMOAD) to halt disease progression, restore structure, and relieve symptoms, there are yet no approved treatments for OA [21–38, 39]. The reason, at least in part, is the heterogeneous nature of OA, which makes it difficult to identify subtypes of the disease, delineate its rate of progression, or anticipate responsiveness to drugs or treatments. Identifying effective biomarkers for Burden of disease, Investigative, Prognostic, Efficacy of intervention, and Diagnostic– (BIPED) purposes is essential for targeting tailored and effective treatment of OA patients [21–38, 39]. Diagnosing OA at early stages and assessing the rate of progression may better support drug development and contribute to the establishment of efficacious treatment regimens that may be adapted to each OA patient [18].

As there are no effective therapies for OA, we here opted to assess biomarkers using a cohort of patients subjected to KJD surgical procedures [30–32, 40, 41, 42]. This method is reported extensively and is accompanied by measurable molecular response in SF to joint distraction (6-week procedure), which appears to be associated with patient-reported outcomes [39], and increased cartilage thickness after 12 months post-distraction [29–31, 43, 41, 44]. This method not only halts damage but also promotes cartilage anabolism and restores its structure.

Since SIRT1 activity was associated with cartilage and joint integrity, we attempted to examine if it may serve as a biomarker for therapeutic response using KJD cohorts. A previous study from our groups identified increased serum levels of NT/CT SIRT1 in association with moderate OA severity in mice and human serum samples. However, this method did not detect variations between moderate to severe OA for human samples [29]. Testing NT/CT SIRT1 for the KJD cohort, we were unable to observe a clear indication of responsiveness, as would be expected by a reduction in the biomarker in association with the two-year structural changes. However, in line with previous work, whereby the increase in NT/CT SIRT1 had indicated more histological or endoscopic cartilage damage [29], here, the increase of NT/CT SIRT1 was associated with increased osteophytes, and PIIANP/CTX-II, at baseline. These data indicate that the emergence of NT/CT SIRT1 fragments are likely involved in processes of inflammation and damage, as can be observed at baseline. On the other hand, the same NT/CT SIRT1 increase was also positively correlated with more structural improvement, which is conceptually contradictory and insinuates that NT/CT SIRT1 is not a suitable measure to reflect post-distraction structural response of cartilage measures.

Contrarily, in this first-time analysis of our novel flSIRT1 sandwich ELISA method, we detected upward trends of serum flSIRT1 accompanied with increased variables of cartilage structural improvement post-distraction. Among all the structural variables, we noted that cartilage-related improvement in dABp (%) or reduced denuded bone exhibited increased trends for serum flSIRT1. The increased trend of serum flSIRT1 was coupled with improved MRI-associated structural measures, which is in line with lesser SIRT1 cleavage and maintenance of epigenetic SIRT1 activity, promoting articular cartilage integrity, as previously reported [7–23–26, 27–29].

## Conclusions

Our results display at the baseline higher NT/CT SIRT1, which were significantly correlated with osteophytes and impaired collagen type II turnover, potentially reflecting baseline joint damage related to OA severity. However, post-KJD cartilage-related structural improvement displayed a similar upward trend for NT/CT SIRT1 in relation to structural improvement, which does not support the use of this biomarker for KJD response. The new ELISA formulation detecting flSIRT1, while limited, exhibited an upward trend of serum flSIRT1 coupled with patient responsiveness and improvement in dABp, 2-year post-distraction. Notably, we did not reach statistical significance for flSIRT1 which may be a result of the method’s technical limitations, restricted patient number, and response heterogeneity.

Overall, NT/CT SIRT1 exhibits baseline positive association with osteophytes and reduced PIIANP/CTXII, reflecting OA severity at baseline. On the other hand, flSIRT1 exhibits a consistent positive trajectory accompanied with a structural response and reduced denuded bone, albeit statistcally insignificant. While future studies must be undertaken to improve the assay and examine larger cohorts, these data are encouraging, and provide support of a potential biochemical endotype reflecting responsiveness to distraction procedure.

### Electronic supplementary material

Below is the link to the electronic supplementary material.

Supplementary Material 1

## Data Availability

The datasets generated during and/or analyzed during the current study are available in the article and supplementary files. Additional data may be obtained upon reasonable request to both the authors MDG (corresponding) and SCM.

## Abbreviations

MRI: Magnetic Resonance Imaging
BMP: Bone morphogenic protein
TGF: Transforming Growth Factor
WNT: Wingless and Int-1
IL1β: Interleukin 1Alpha
TNFα: Tumor necrosis factor ALPHA
SIRT1: Sirtuin 1
KJD: Knee joint distraction
OA: Osteoarthritis
75SIRT1: 75kD fragment
mCT: monoclonal CT-specific denoted
pNT: Polyclonal NT-specific denoted
NT: N terminal
CT: C terminal
Fl: Full Length
BMI: Body mass index
KOOS: Knee injury and osteoarthritis outcome score
VAS: Visual analogue scale
MAC: Most affected compartment
ThCtAB: Mean cartilage thickness over the total subchondral bone area
DABp: Percentage of denuded subchondral bone area
JSW: Joint space width
NT/CT: N-terminal/C-terminal
flSIRT1: Full-length sirtuin-1
PIIANP: N-propeptide of collagen IIA
CTX-II: C-terminal cross-linked telopeptides of type II collagen
RCT: Randomized Control trial

## Declarations

### Animal studies

Not Applicable to this report.
