# Supplementary material for: Two-year post-distraction cartilage-related structural improvement is accompanied by increased serum full-length SIRT1
Source: Arthritis Res Ther. 2024 May 24;26:106. doi: 10.1186/s13075-024-03342-5 (PMC11127335; doi:10.1186/s13075-024-03342-5)
Supplement: Supplementary file 1 — Supplementary Material 1 [file 13075_2024_3342_MOESM1_ESM.docx]

| **1** | **%CV inter-plates** | 4.76% ± 1.22 | |
| --- | --- | --- | --- |
| **2** | **%CV intraplate** | 10.84% ± 0.02 | |
| **3** | **Spike in** | 81.63% ± 37.31 | |
| **4** | **Dilution recovery** | 80.18% ± 47.80 | |
| **5** | **Antibody** | Capture: mCT2-monoclonal antibody | Detection: pNT1-polyclonal antibody |
| **6** | **Antibody epitope** | Human SIRT1 C-terminus (~530-747bp) | Human SIRT1 N-terminus (~10-150bp)‎ |

‎

**SD1: Antibody validation for flSIRT1 ELISA sandwich detection**: **Row 1 and 2**: Percent coefficient of variance (%CV) was calculated for flSIRT1 ELISA sandwich‎ using human serum ‎samples, in four identical plates. The mean concentration for a given ‎sample was determined and divided by the standard deviation. Values were ‎then multiplied by 100 to achieve %CV values between 4 plates (Inter-plates) or within 4 wells in a given plate (intraplate). **Row 3:** ‎Spike in: Neat serum samples were diluted 1:2000 in PBS and spiked with ‎‎‎100ng/mL flSIRT1‎ (i.e. denoted "spiked" value). Using the standards curve, we calculated the spiked and unspiked values, which were subtracted and devided by the expected value (i.e. the sum of separate values for spike and unspiked samples). The result was multiplied by 100 to reach % spiked in recovery. The closer the value to 100%, the best the reflects the concentration of the Spiked sample. **Row 4**: Dilution recovery for flSIRT1 ELISA sandwich, was calculated for ‎different serum samples, each individually diluted 1:2000 or 1:3000 ‎, which is within the working dilution range. Dilution recovery was ‎calculated by ((A)/(B*2))*100. Last two rows in table include ‎epitope ‎recognition for both generated antibodies pNT1 (detection) and mCT2 (capture).
